# Glucagon-like peptide-1 receptor agonists in orthopaedics

**DOI:** 10.1051/sicotj/2025067

**Published:** 2026-03-06

**Authors:** Andreas F. Mavrogenis, Pavlos Altsitzioglou, Sotirios Pililis, Athanasios D. Anastasilakis, Symeon Tournis, Polyzois Makras, Theodosis Saranteas, Sebastien Lustig

**Affiliations:** 1 First Department of Orthopaedics, National and Kapodistrian University of Athens, School of Medicine Athens 11527 Greece; 2 Diabetes Center, Second Department of Internal Medicine, National and Kapodistrian University of Athens, School of Medicine Athens 11527 Greece; 3 Department of Endocrinology, Diabetes and Metabolism, 424 General Military Hospital Thessaloniki 56429 Greece; 4 Laboratory for Research of the Musculoskeletal System “Th. Garofalidis”, National and Kapodistrian University of Athens, School of Medicine Athens 14561 Greece; 5 Department of Endocrinology and Diabetes, Department of Medical Research, 251 Hellenic Air Force General Hospital Athens 11525 Greece; 6 Second Department of Anesthesiology, National and Kapodistrian University of Athens, School of Medicine Athens 11527 Greece; 7 Orthopaedics Surgery and Sports Medicine Department, FIFA Medical Center of Excellence, Croix-Rousse Hospital, Hospices Civils de Lyon, Lyon North University Hospital Lyon 69004 France; 8 University of Lyon, Claude Bernard Lyon 1 University, IFSTTAR Lyon 69622 France

**Keywords:** GLP-1, Orthopaedic surgery, Bone mineral density, Wound healing

## Abstract

Glucagon-like peptide-1 (GLP-1) receptor agonists (GLP-1RA) help people control blood glucose and lose weight. They may also help with bone metabolism, healing fractures, keeping joints healthy, and recovering after surgery. There is growing amount of evidence of their ability to modulate the activity of osteoblasts and osteoclasts, affect inflammatory pathways, and interact with neuroprotective and psychological systems. Although the growing importance of GLP-1 receptor agonists in orthopaedics marks a major shift in how metabolic medicines affect musculoskeletal health, current knowledge is still basic and lacks information on long-term results, safety, and how well different treatments work compared to one another. This paper summarizes the existing evidence on the effects of GLP-1RA drugs on bone metabolism and healing, and discusses their role in current orthopaedics.

## Introduction

Glucagon-like peptide-1 (GLP-1) is an amino-acid peptide hormone deriving from posttranslational processing of the proglucagon peptide, and produced and secreted by intestinal enteroendocrine L-cells and certain neurons within the nucleus of the solitary tract in the brainstem upon food consumption. It enhances the secretion of insulin; therefore, it decreases blood sugar levels in a glucose-dependent manner, and it has been associated with numerous regulatory and protective effects, including obesity and bone metabolism.

The introduction of glucagon-like peptide-1 receptor agonists (GLP-1RA) into clinical practice in the 2000s has revolutionized the management of obesity and type 2 diabetes mellitus. Initially developed as antidiabetic medications, currently available GLP-1RA, including semaglutide, liraglutide, exenatide, and dulaglutide, represent the most widely used medications for weight loss. In orthopaedics, current evidence suggests an important role of these drugs in homeostasis of bone metabolism and orthopaedic recovery [1, 2]. However, even if this evidence is promising, the link between GLP-1 and bone health is still unclear [2], and further research is necessary to clarify the long-term impacts of GLP-1RA on bone health. Moreover, the increasing use of these drugs has led to specific anesthesiology guidelines and considerations for patients undergoing surgery while on GLP-1RA treatment.

Therefore, we performed this editorial to summarize existing evidence on the effects of GLP-1RA drugs on bone metabolism and healing, to discuss their current role in orthopaedic applications, and to emphasize the anesthesia considerations for patients being administered GLP-1RA undergoing orthopaedic surgery.

## GLP-1RA in orthopaedic surgery, recovery, and rehabilitation

GLP-1RA offers multiple advantages that may enhance recovery protocols and facilitate improved patient recovery after orthopaedic surgery, particularly for individuals with comorbid conditions that complicate the recovery process [3–10]. In addition to their direct effects on bone tissue, GLP-1RA may provide systemic benefits that directly promote anti-inflammation and indirectly promote orthopaedic recovery [9, 10]. Recent evidence suggests that GLP-1RA exert anti-inflammatory effects, which may help reduce the incidence of complications such as delayed union, surgical site infections, and poor wound healing, as well as to provide for pain relief and improved mobility after surgery, rehabilitation, and return to normal activities [3, 4]. By modulating pro-inflammatory cytokines that are known to slow down the healing and growth of bones, and improving systemic metabolic status, GLP-1RA could contribute to a more favorable healing environment during the perioperative and postoperative phases of orthopedic care [1, 5].

The relationship between GLP-1RA and diet may be an important area of study because improving nutrition while on GLP-1 medication could positively affect the metabolic and bone health, and create a more favorable environment for healing, which will lead to better recovery and outcomes for orthopaedic patients. In this context, it will be very helpful to customize GLP-1RA therapy to fit the needs of each patient, especially those who have other metabolic diseases or a specific history of surgery. For instance, understanding the different inflammatory and metabolic responses in obese or diabetic patients may help doctors better adjust the timing and dosage of GLP-1RA in relation to surgery, which could improve both metabolic regulation and the ability of bones to heal [11, 12].

Additionally, GLP-1RA may have a substantial impact on the psychological aspects of rehabilitation, especially in people who are dealing with worry and depression that prevail after orthopaedic surgery. Combining GLP-1 therapy with mental health support may lead to a more holistic approach to patient care, creating a complete recovery plan that includes both the body and the mind [6]. In this respect, GLP-1RA has shown a protective role in the nervous system [7]. They can cross the blood-brain barrier and interact with GLP-1 receptors in the central nervous system. This can improve neuroprotection and cognitive function, which are especially helpful for people with postoperative delirium or cognitive decline, elderly patients, and those with comorbidities [7]. Adding GLP-1RA therapy to postoperative care plans could enhance physical recovery and maintain cognitive function.

## GLP-1RA in bone homeostasis

**Figure 1:**
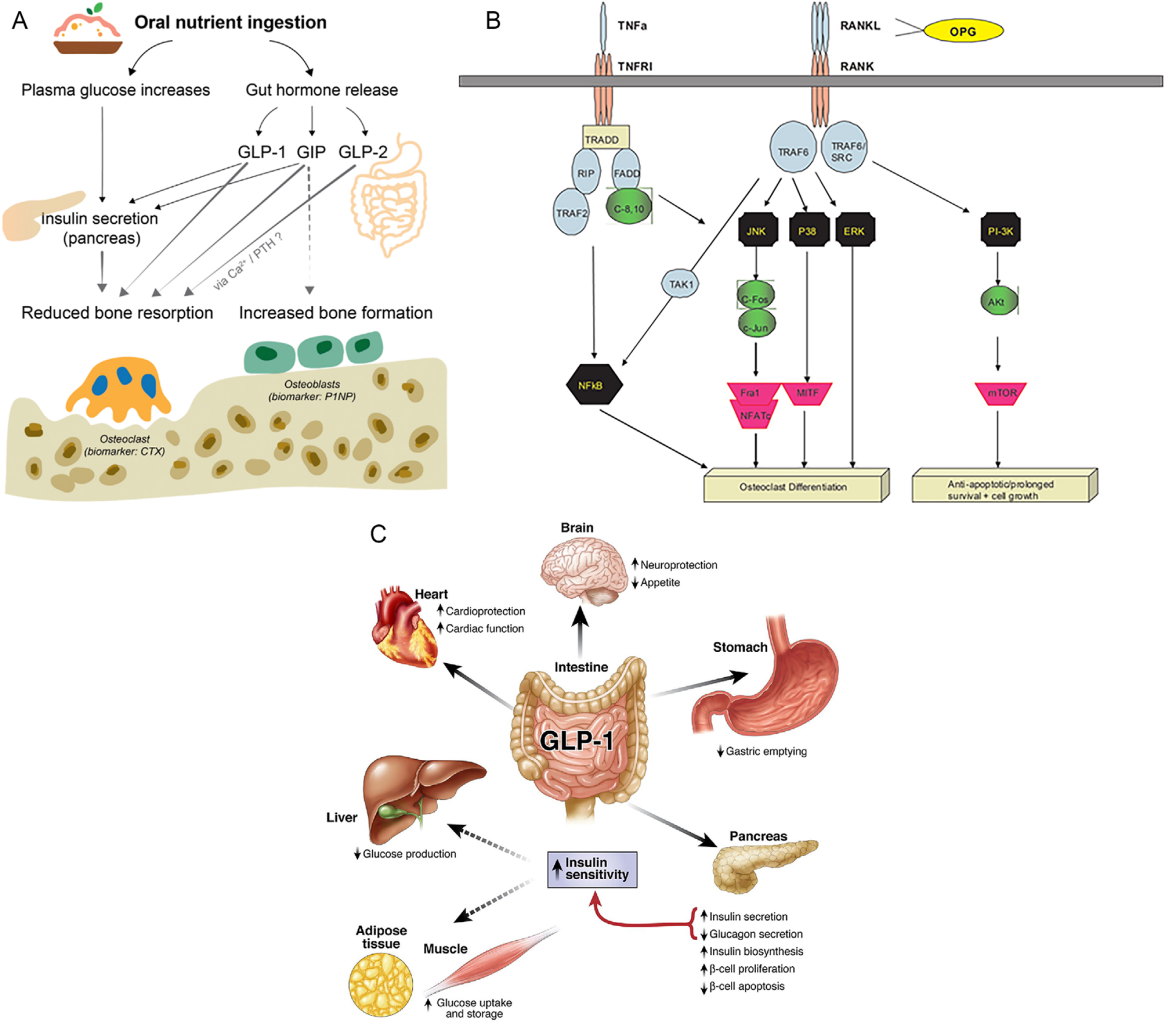
(A) Post-prandial gut release of GLP-1RA and downstream reduction in bone resorption (CTX) alongside increased osteoblast activity (P1NP). (B) Key nodes of the OPG/RANKL/RANK pathway that GLP-1RA may modulate to limit osteoclastogenesis. (C) System-wide actions of GLP-1RA (weight loss, improved insulin sensitivity) that indirectly enhance the peri-operative bone micro-environment.

GLP-1RA exerts multifaceted effects on bone metabolism by interacting with several key signaling pathways involved in bone remodeling (Figure 1; Table 1). One of the most notable is the Wnt/β-catenin pathway, which plays a central role in osteoblast differentiation, proliferation, and function. GLP-1 has been shown to enhance this pathway, potentially through downregulation of sclerostin, a glycoprotein that inhibits Wnt signaling and limits bone formation [1]. By reducing sclerostin levels, GLP-1RA may stimulate new bone formation and improve bone quality; this effect is especially important in patients suffering from conditions such as osteoporosis or delayed fracture healing.

**Table 1:** Mechanistic axes of GLP-1RA action relevant to orthopaedics.

Study	Mechanistic axes	Principal effects	Comments
Luo et al. [1]	↓ Sclerostin → ↑ Wnt/β-catenin; RANKL/OPG modulation; Anti-inflammatory cytokine shift	Enhances osteoblastogenesis, reduces osteoclastogenesis, and promotes a healing milieu	Preclinical studies: improved bone mass/strength; relevant in osteoporosis and fracture healing
Spence et al. [4]	Weight loss & metabolic improvements	Reduces joint load, lowers complication risk	Indirect musculoskeletal benefit, but weight loss may reduce BMD
Anastasilakis et al. [8]	Dual/triple incretin agonists; Amylin analogues; ActRII antagonists	Stronger anabolic potential; bone & muscle preservation	Novel therapies may improve skeletal outcomes
Nance [19]	Chondrocyte/cartilage homeostasis	May slow osteoarthritis progression, improve joint function	Preclinical chondroprotection, relevant in knee osteoarthritis

OPG: osteoprotegerin; BMD: bone mineral density; ActRII: Activin type II receptor.

Similarly, GLP-1 appears to influence osteoclastogenesis by modulating the RANK/RANKL/OPG signaling axis. Through increasing osteoprotegerin (OPG) or decreasing RANKL expression, GLP-1RA can inhibit the formation and activity of osteoclasts, thereby reducing bone resorption. This dual action (promoting osteoblast function while suppressing osteoclast activity) suggests that GLP-1RA may support a more balanced and favorable bone remodeling environment and enhance bone homeostasis. This could be particularly valuable for individuals at increased risk of fragility fractures, including elderly patients and those with comorbid metabolic diseases. The GLP-1RA liraglutide and exenatide have been shown to promote osteoblast proliferation, enhance differentiation of mesenchymal stem cells toward osteoblasts, and inhibit osteoclast formation. These effects, while promising, are dose-dependent and often observed at pharmacologic concentrations significantly higher than those used in clinical practice [8]. Moreover, clinical evidence regarding bone turnover markers in patients under GLP-1RA therapy is inconsistent. Some increases in bone turnover markers may be attributed to weight loss itself rather than a direct pharmacological effect [8].

## GLP-1RA in osteoporosis

GLP-1RA optimizes metabolic processes and reduces inflammation [13]. Clinical findings on bone mineral density changes following GLP-1RA therapy are mixed; while some studies report reductions in lumbar spine and hip bone mineral density, other studies show stability or minor increases [1, 8, 13–15]. These effects often correlate with the degree of weight loss. Importantly, combining GLP-1RA with weight-bearing exercise may mitigate bone mineral density loss, as seen in the largest study where liraglutide plus exercise maintained bone mineral density despite significant weight reduction [8]. Moreover, GLP-1RA can enhance osteogenic differentiation while inhibiting adipogenesis. This indicates its potential utility in addressing obesity-related issues that impact bone health [1].

Meta-analyses provide mixed but generally favorable evidence regarding the impact of GLP-1RA on fracture risk. Liraglutide appears to be the most studied agent and is often associated with reduced fracture risk in type 2 diabetes mellitus patients. Exenatide has yielded conflicting outcomes, being linked to both lower and higher fracture risk in separate analyses, emphasizing the need for further comparative trials [8].

## GLP-1RA in fracture healing

GLP-1RA correct the metabolic imbalance that is common in people with type 2 diabetes. The combination of high blood sugar and inflammation can make bones weaker and more likely to break [16]. In this respect, GLP-1RA may make it easier for bones to heal and remodel by lowering systemic inflammation and controlling blood sugar levels. This could lead to better outcomes in postoperative fracture care. Understanding how GLP-1 signaling interacts with other hormonal pathways involved in bone metabolism, like the Wnt/β-catenin pathway, could lead to new ways to treat bone fractures. Also, the fact that GLP-1RA can alter the gut-bone axis unlocks a lot of interesting study prospects, especially when it comes to figuring out how gut microbiome and GLP-1 signaling affect bone health. Recent research shows that the gut microbiome has a big effect on systemic inflammation and metabolic processes, which are important for optimal bone remodeling and recovery after surgery [17]. Physicians may be able to improve the therapeutic benefits for patients with metabolic issues, especially those having orthopedic surgery, by studying how GLP-1RA drugs work together with gut health programs. This integrative approach shows how important it is to have a holistic therapeutic model and how important it is to use personalized strategies that take into account the differences in gut flora between people. This could lead to better results in bone healing and overall recovery. Yet, despite concerns regarding bone turnover and bone mineral density, large-scale studies such as the SELECT trial have shown no significant difference in overall fracture incidence between GLP-1RA and placebo groups. However, a numerically higher fracture rate for hip and pelvic fractures was observed among older women, warranting caution in high-risk populations [8].

## GLP-1RA in arthritis

Ongoing research shows that GLP-1RA has a complicated role in both the metabolic and mechanical aspects of joint disorders. GLP-1RA may assist in managing joint problems, particularly osteoarthritis, potentially due to their anti-inflammatory effect [18]. GLP-1RA can manage the metabolic environment of cartilage and make cartilage more flexible [18]. Recent research suggests that GLP-1RA may affect the function of chondrocytes and the balance of cartilage that could help slow down the degenerative processes of osteoarthritis [19]. GLP-1RA drugs may create an anabolic environment in the joint that could reduce symptoms and slow the disease’s progress. GLP-1RA may improve the viscoelastic properties of synovial fluid, therefore, enhancing its ability to absorb shocks and lubricate the joint. Other studies have shown that GLP-1RA makes insulin more effective in addition to lowering the body mass index, providing for joint health by putting less stress on weight-bearing joints and lowering inflammatory markers connected to metabolic syndrome [20].

## Safety profiles of GLP-1RA in orthopaedic patients

There are concerns about gastrointestinal side effects, especially nausea and vomiting, with the use of GLP-1RA in orthopaedic surgery, especially during recovery from surgery [21]. Several clinical studies have shown that the likelihood of more adverse events is higher, which shows how important it is to have tailored treatment plans that consider each patient’s unique health condition and risk factors [22]. Physicians should weigh the metabolic and skeletal benefits of GLP-1RA drugs in orthopaedic patients against a full grasp of the safety risks. This will encourage a patient-centered strategy that focuses on both effectiveness and well-being (Table 2).

**Table 2:** Clinical evidence of GLP-1RA relevant to orthopaedic outcomes.

Study	Orthopaedic/surgical contexts	Key findings	Comments
Driessen et al. [2]	Population-based T2DM cohorts	No correlation between GLP-1RA use and fracture incidence	Fracture risk neutral overall
Wiener et al. [5]	Spine surgery (15,000 patients)	Improved rehab engagement, fewer recovery barriers	Demonstrates holistic recovery benefit
Anastasilakis et al. [8]	RCTs & cohorts in T2DM & PwO	Mixed BMD results; LS stable, TH ↓; BTMs ↑; fracture risk neutral; possible hip-specific benefit	Exercise mitigates BMD loss; weight loss confounder
Mabilleau [16]	Bone quality & fracture healing in T2DM	GLP-1RA stabilises glucose/inflammation → better healing	Mechanistic support for metabolic-bone axis
Magaldi et al. [21]	Total hip arthroplasty	Reduced postoperative complications, GI side-effects noted	Feasible in arthroplasty setting
Kaneko et al. [30]	Perioperative glycemic control in ERAS	Liraglutide improved metabolic stability peri-op	Associated with reduced risk of cardiovascular events and infections, better weight management and better recovery

T2DM: type 2 diabetes mellitus; RCTs: randomized controlled trials; PwO: people living with obesity; ERAS: enhanced recovery after surgery; BMD: bone mineral density; LS: lumbar spine; TH: total hip; BTM: bone turnover markers.

## Anesthesia considerations in orthopaedic patients receiving GLP-1RA

Recommendations for managing patients taking glucagon-like peptide-1 receptor agonists (GLP-1RAs) and dual GLP-1/glucose-dependent insulinotrophic peptide receptor co-agonists (GLP-1/GIPRAs) prior to anaesthesia or sedation and subsequently prior to surgery are of fundamental clinical importance [1]. In fact, retained gastric contents and potential pulmonary aspiration during general anesthesia or sedation in diabetes and/or obesity treated patients have been reported [23, 24]. However, little evidence exists to determine the best approach to managing these therapeutics perioperatively. In particular, adhering to the American Society of Anesthesiologists guidelines [25], withholding daily formulations on the day of surgery, and weekly formulations a week prior to surgery are strongly recommended. Nevertheless, specific parameters warrant careful consideration.

Physicians must identify patients receiving GLP-1RA therapy who simultaneously present a high risk of aspiration. In the following circumstances, further attention is required, and potential hazards leading to potential side effects (aspiration pneumonia) must be properly addressed [26, 27]. Specifically, a) The escalation phase of GLP-1RA treatment (four to eight weeks) is of higher risk of delayed gastric emptying than the maintenance period; b) The higher doses of GLP-1RA possess increased risks of aspiration during surgery; c) Gastrointestinal side effects are more frequently observed after the weekly versus the daily formulations; d) Symptoms such as nausea, vomiting, abdominal pain, dyspepsia, and constipation are warnings of a delayed gastric emptying, although not always reliable indicators of protracted intestinal transit times; e) Other diseases (bowel dysmotility, gastroparesis, and Parkinson’s disease) or medications (chronic pain therapy with opioids) can also delay gastric emptying; and f) The duration of inhibition of gastric emptying from longer acting GLP-1RA and GLP-1/GIPRAs is unknown, especially if long elimination half-life medications are prescribed (Table 3). Therefore, patients suffering from diabetes mellitus should be managed cautiously, because the discontinuation of a GLP-1RA medication for extended periods may result in poor glycemic control during the perioperative period.

**Table 3:** GLP-1RA drugs characteristics.

Drug	Type of receptor agonism	Elimination half-life	Dose interval	Trade name
Liraglutide	GLP-1	12.6–14.3 h	Once daily	Victoza, Saxenda
Tirzepatide	GLP-1 and GIP	4.2–6.1 days	Once weekly	Mounjaro
Dulaglutide	GLP-1	4.7–5.5 days	Once weekly	Trulity
Samaglutide	GLP-1	5.7–6.7 days	Once weekly	Ozempic

GLP-1: Glucagon-like peptide-1; GIP: glucose-dependent insulinotropic peptide.

The critical need for aspiration prophylaxis in patients undergoing surgery while on GLP-1RA therapy is well established. Additionally, measures to mitigate potential adverse events are imperative and may include the following [26–28]: a) For at least a 24-hour clear liquid diet (water, black coffee/tea without milk/creamer, clear juice without pulp, electrolyte drinks) that should be ceased at least two hours prior to surgery, is recommended for populations with high risk of aspiration; b) Routine medication may also be received (with a small amount (30 mL) of water) up to two hours before any procedure; c) A single dose of 3 mg/kg (maximum dose of 250 mg) erythromycin intravenously can be given to facilitate gastric emptying within 15 min; metoclopramide could also be an option of diabetic gastroparesis (10 mg intravenously prior to anesthesia induction); d) Gastric ultrasound (if available) should be performed to determine gastric content and to assess aspiration risk; and e) When concerns persist regarding retained gastric content or if the patient does not conform to clear liquid diet instructions or when urgent/emergency surgery is scheduled, anesthesiologists are advised to treat patients as though they have a full stomach and implement full-stomach precautions.

When contextualizing the management of patients receiving GLP-1RA therapy, therefore, a multidisciplinary approach is essential before surgery. Anesthesiologists, surgeons, and prescribing physicians must collaborate effectively to ensure patients’ safety without unnecessarily extending the interruption of the GLP-1RA medication’s therapeutic benefits.

## Future directions in GLP-1RA research in orthopaedics

Emerging combination therapies involving GLP-1RA are promising future research areas in orthopaedic research. The goal for physicians is to use the many benefits of GLP-1RA drugs to get the best results after surgery and help people make long-lasting health habits that improve their quality of life by creating an environment that encourages these changes. It would be helpful to look at the possible synergistic benefits of GLP-1RA when it is used with other drugs, especially those that treat pain and inflammation. This could help with the study of GLP-1RA in orthopaedics. Combining GLP-1RA with non-steroidal anti-inflammatory drugs may make pain relief more effective while lowering the gastrointestinal side effects that are often associated with long-term NSAID use, especially in postoperative care settings [29]. Also, understanding how GLP-1RAs interact with different metabolic pathways could help make new combination drugs that not only relieve pain but also enhance recovery by improving metabolic health and lowering the risk of complications.

Accelerating metabolic recovery after surgery, particularly in diabetic patients, with the use of GLP-1RA may prove beneficial in orthopaedic surgery [5, 30]. Furthermore, comprehending the interaction of GLP-1 medication with different metabolic treatments may yield novel approaches to address both immediate recovery requirements and sustained health advantages. This underscores the need to adopt a multidisciplinary strategy in the management of patients undergoing orthopedic surgery. In this respect, cooperation between endocrinologists, orthopaedic surgeons, and rehabilitation specialists in order to create comprehensive care plans that make the most of the many benefits of GLP-1 therapies should be encouraged. This way of working together could lead to new ways of treating patients. Increasing research indicates that GLP-1RA drugs may be integral to full healing protocols; therefore, their numerous roles in patient care should be investigated further.

## Data Availability

The literature research data related to the present article are available from the corresponding author upon request.
